# Vitamin D3 Stimulates Proliferation Capacity, Expression of Pluripotency Markers, and Osteogenesis of Human Bone Marrow Mesenchymal Stromal/Stem Cells, Partly through SIRT1 Signaling

**DOI:** 10.3390/biom12020323

**Published:** 2022-02-18

**Authors:** Ana Borojević, Aleksandra Jauković, Tamara Kukolj, Slavko Mojsilović, Hristina Obradović, Drenka Trivanović, Milena Živanović, Željko Zečević, Marija Simić, Borko Gobeljić, Dragana Vujić, Diana Bugarski

**Affiliations:** 1 Mother and Child Health Care Institute of Serbia ‘’Dr Vukan Čupić’’, 11000 Belgrade, Serbia; zeljko.zecevic75@gmail.com (Ž.Z.); makata913@gmail.com (M.S.); borko.gobeljic@gmail.com (B.G.); vujicdbg@gmail.com (D.V.); 2 Group for Hematology and Stem Cells, Institute for Medical Research, National Institute of Republic of Serbia, University of Belgrade, 11129 Belgrade, Serbia; aleksandra@imi.bg.ac.rs (A.J.); tamara.kukolj@imi.bg.ac.rs (T.K.); slavko@imi.bg.ac.rs (S.M.); hristina.obradovic@imi.bg.ac.rs (H.O.); drenka.trivanovic@gmail.com (D.T.); milenaziv97@gmail.com (M.Ž.); dianab@imi.bg.ac.rs (D.B.); 3 IZKF Group Tissue Regeneration in Musculoskeletal Diseases, University Clinics, Röntgenring 11, 97070 Würzburg, Germany; 4 Bernhard-Heine-Center for Locomotion Research, University Würzburg, Sanderring 2, 97070 Würzburg, Germany; 5 School of Medicine, University of Belgrade, 11000 Belgrade, Serbia

**Keywords:** bone marrow mesenchymal stromal cells (BM-MSCs), vitamin D3 (cholecalciferol, VD3), SIRT1, regenerative potential, stemness, osteogenesis

## Abstract

The biology of vitamin D3 is well defined, as are the effects of its active metabolites on various cells, including mesenchymal stromal/stem cells (MSCs). However, the biological potential of its precursor, cholecalciferol (VD3), has not been sufficiently investigated, although its significance in regenerative medicine—mainly in combination with various biomaterial matrices—has been recognized. Given that VD3 preconditioning might also contribute to the improvement of cellular regenerative potential, the aim of this study was to investigate its effects on bone marrow (BM) MSC functions and the signaling pathways involved. For that purpose, the influence of VD3 on BM-MSCs obtained from young human donors was determined via MTT test, flow cytometric analysis, immunocytochemistry, and qRT-PCR. Our results revealed that VD3, following a 5-day treatment, stimulated proliferation, expression of pluripotency markers (NANOG, SOX2, and Oct4), and osteogenic differentiation potential in BM-MSCs, while it reduced their senescence. Moreover, increased sirtuin 1 (SIRT1) expression was detected upon treatment with VD3, which mediated VD3-promoted osteogenesis and, partially, the stemness features through NANOG and SOX2 upregulation. In contrast, the effects of VD3 on proliferation, Oct4 expression, and senescence were SIRT1-independent. Altogether, these data indicate that VD3 has strong potential to modulate BM-MSCs’ features, partially through SIRT1 signaling, although the precise mechanisms merit further investigation.

## 1. Introduction

Bone marrow mesenchymal stromal/stem cells (BM-MSCs) were initially discovered as a non-hematopoietic fraction of the bone marrow niche, capable of fully reconstructing the hematopoietic microenvironment [1]. Further studies have recognized BM-MSCs as a cell population with distinctive characteristics, such as multilineage differentiation capacity, trophic paracrine activity, control of inflammation, and homing to sites of injury, all of which nominate them as prospective tools in regenerative medicine [2]. Although different MSCs have been discovered in virtually all tissues, BM-MSCs represent the most thoroughly studied type of these cells with prevalence in clinical studies [3,4]. As for bone tissue repair, BM-MSCs’ therapeutic potential has been associated with their capacity to directly differentiate into cells of osteogenic lineage, as well as to generate an appropriate regenerative microenvironment via secretion of various growth factors [5]. Considered to be a cornerstone of bone tissue engineering, the application of BM-MSCs has been combined with various types of biomaterials to facilitate the ossification process and improve clinical therapy of delayed bone healing—usually related to various bone diseases, injuries, or tumor surgery [5]. Some of the recent advances that have emerged with an aim to develop more effective therapies for enhanced bone regeneration propose loading of different hybrid biomaterial matrices or 3D scaffolds with vitamin D3 [6,7,8,9].

Vitamin D3, or cholecalciferol, is well known for its role in the regulation of calcium and phosphorus balance, which contributes to bone homeostasis and strength. In addition to stimulating the intestinal absorption of the aforementioned minerals, along with reducing their renal excretion, vitamin D3 enhances bone calcification and stimulates various osteoblast functions, such as differentiation, proliferation, and expression of specific proteins and growth factors [10]. Vitamin D3 is generated in the skin from 7-dehydrocholesterol via a process that involves UV light exposure, or can be obtained through diet or supplements. Biologically active metabolites of vitamin D3 are generated through a two-step hydroxylation process that includes its conversion to 25-hydroxyvitamin D3 (25(OH)D3, or calcidiol) in the liver by cytochrome enzymes—25-hydroxylases (CYP2R1 and CYP27A1)—and then in the kidneys to 1α,25-dihydroxyvitamin D3 (1α,25(OH)_2_D3 or calcitriol) by 1α-hydroxylase (CYP27B1) [11]. In addition, another 24-hydroxylase (CYP24A1) contributes to vitamin D3 homeostasis through inactivation of 25(OH)D3 and 1α,25(OH)_2_D3 [12]. Apart from the kidneys, many other cells have been shown to express 1α-hydroxylase, including keratinocytes, various immune cells, and cancer cells [13].

Several studies have confirmed that BM-MSCs and adipose tissue MSCs also possess vitamin-D-metabolizing machinery consisting of vitamin D receptors (VDRs), 25-hydroxylase, 1α-hydroxylase, and 24-hydroxylase [14,15,16,17]. Moreover, in vitro studies have demonstrated stimulatory effects of the vitamin D3 metabolite 1α,25(OH)_2_D3 on BM-MSCs’ osteogenic differentiation, as well as of 25(OH)D3, depending on 1α-hydroxylase activity in the cells [15,18]. These findings suggest an autocrine/paracrine role of vitamin D metabolism in BM-MSCs’ osteogenesis. However, the influence of the vitamin D3 precursor cholecalciferol (VD3) on MSCs’ properties remains largely unknown, although its modulatory effect on osteoblasts’ proliferation—but not differentiation—has been reported [19]. In addition, recent investigations utilizing various hybrid biomaterial scaffolds or composites loaded with VD3 have demonstrated their enhanced biocompatibility and/or osteoinductive effects on MSCs, implying the advantages of their use for the treatment of bone defects. Specifically, a stimulatory effect on murine BM-MSCs’ viability and proliferation rate was demonstrated for a VD3-loaded composite cement scaffold containing calcium sulfate and calcium citrate, which enabled sustained and controllable release of this vitamin [7]. In addition, polycaprolactone/gelatin scaffolds loaded with VD3 in combination with nano-hydroxyapatite (HAP) stimulated osteogenesis of adipose tissue MSCs, increasing the ALP activity in the early stages and mineralization in the late stages of differentiation [6]. Similarly, an osteoinductive effect was demonstrated for human BM-MSCs grown on VD3/vitamin K2/Mg-loaded biodegradable polymer nanofibers [20], as well as on human dental pulp MSCs seeded on chitosan scaffolds constructed to sustainably deliver VD3 over the course of 3–5 days [21].

Although these studies indicate that VD3 in combination with various biomaterial matrices exerts pro-osteogenic effects on different types of MSCs, the mode of VD3 action per se on the MSCs’ regenerative potential has not been studied before. In order to better define VD3′s potential for use as a pretreatment factor in stem-cell-based regenerative medicine, we aimed to analyze whether and how VD3 modulates stem cell features of human BM-MSCs. For that purpose, proliferation capacity, osteogenic differentiation potential, and the expression of pluripotency-, differentiation-, and senescence-associated markers were investigated in young donors’ BM-MSCs after 5-day treatment with VD3. Moreover, in order to elucidate the mechanisms underlying VD3′s effects on BM-MSCs, the involvement of SIRT1 (sirtuin 1; silent mating type information regulation 2 homolog 1) was investigated, given the well-documented regulatory role of this signaling molecule in MSCs’ self-renewal and differentiation [22].

## 2. Material and Methods

### 2.1. Isolation and Cultivation of BM-MSCs

Bone marrow samples were obtained at the Mother and Child Health Care Institute of Serbia. At the time of collection for allogenic transplantation, 2 mL of bone marrow was aspirated from an iliac bone for the purpose of this research. Informed consent was provided for the collection of samples from 5 healthy donors (2–12 years old). All samples were collected in accordance with the local ethical committee’s standards and the Declaration of Helsinki.

Upon collection, a fraction of mononuclear cells (MNCs) was isolated using lymphocyte separation medium (Lymphocyte Separation Medium 1077, Capricorn Scientific, Ebsdorfergrund, Germany) and density gradient centrifugation. Isolated MNCs were further cultivated in growth medium (GM) in plastic tissue culture flasks (Greiner Bio-One, Monroe, NC, USA). GM consisted of MEM Alpha Modification medium supplemented with nucleosides (Capricorn Scientific), 10% fetal bovine serum (FBS, Gibco, Thermo Fisher Scientific, Waltham, MA, USA), 1% penicillin/streptomycin (Gibco, Thermo Fisher Scientific), and 1% L-glutamine (Capricorn Scientific) [23]. Cells were cultured under standard conditions at 37 °C in a humidified atmosphere containing 5% CO_2_, with exchanges of GM two times a week. Non-adherent mononuclear cells were successively removed, and adherent fibroblast-like cells were cultivated until reaching 80–90% confluence, when they were harvested using 0.25% trypsin/EDTA (Capricorn Scientific). After enumeration and viability assessment, cells were re-plated for further passaging at a concentration of 1 × 10^4^ cells/cm^2^. All experiments were conducted using BM-MSCs from passages 2 to 7.

### 2.2. Characterization of BM-MSCs

To confirm that the isolated adherent cells were MSCs, immunophenotype and multilineage differentiation were analyzed following the minimal criteria proposed by The International Society for Cellular Therapy [24].

#### 2.2.1. Immunophenotype Analysis

Surface marker expression on BM-MSCs expanded in vitro was analyzed by flow cytometry as previously described [25]. BM-MSCs cultured under standard conditions were detached using 1 mL of Accutase (Biowest, Nuaillé, France) and washed in cold phosphate-buffered saline (PBS, Capricorn Scientific) with 0.5% bovine serum albumin (BSA, Sigma-Aldrich, St. Louis, MO, USA). Subsequently, BM-MSCs divided into aliquots of 2 × 10^5^ cells were labeled in the dark at 4 °C for 30 min with fluorescein isothiocyanate (FITC)-, phycoerythrin (PE)-, or cyanine 5 (Cy5)-PE-conjugated monoclonal antibodies against human antigens CD44H, CD73, CD90, CD33, CD235a (all from R&D Systems, Minneapolis, MN USA), CD45, CD105, HLA-DR (all from Invitrogen, Carlsbad, CA, USA), and CD34 (DakoCytomation, Glostrup, Denmark). Incubation with corresponding FITC-, PE-, and Cy5-PE-conjugated isotype control antibodies (R&D Systems and Invitrogen) was used to detect the level of nonspecific binding. Flow cytometry was performed using a Cytomics FC 500 cytometer (Beckman Coulter, Brea, CA, USA), while data analyses were accomplished using WinMDI 2.9 software (J. Trotter, The Scripps Research Institute, La Jolla, CA, USA) and NovoExpress 1.2.4 software (Agilent, Santa Clara, CA, USA).

#### 2.2.2. In Vitro Multilineage Differentiation

The differentiation potential of BM-MSCs towards three lineages (osteogenic, chondrogenic, and adipogenic) was also estimated, in order to confirm their functional properties, as previously described [25]. BM-MSCs were seeded in 96-well plates (5 × 10^3^ cells/well) and cultured in GM under standard conditions. At the stage of culture sub-confluence, GM was replaced with corresponding differentiation media (DM).

Induction of osteogenesis was conducted using an osteogenic medium consisting of GM supplemented with 10 mM β-glycerophosphate (AppliChem), 10 nM dexamethasone (Sigma-Aldrich, St. Louis, MO, USA), and 50 μM ascorbic acid-2-phosphate (Sigma-Aldrich). Early osteogenesis was detected after 7 days by alkaline phosphatase (ALP) staining with 5-bromo-4-chloro-3-indolyl phosphate/nitro blue tetrazolium (Sigma-Aldrich), while late osteogenic differentiation was determined after 21 days by Alizarin Red staining of mineralized extracellular matrix (Merck, Darmstadt, Germany).

Chondrogenic differentiation was induced using GM supplemented with 10 nM dexamethasone (Sigma-Aldrich), 2 ng/mL transforming growth factor-β1 (TGF-β1) (R&D Systems), and 50 μM ascorbic acid-2-phosphate (Sigma-Aldrich). To confirm the formation of cartilage-specific glycosaminoglycans, staining with Safranin O (Merck Chemicals, Burlington, MA, USA) was performed after 21 days of cultivation.

For induction of adipogenic differentiation, cells were incubated in GM containing 1 µM dexamethasone (Sigma-Aldrich), 10 µg/mL insulin (Sigma-Aldrich), and 100 µg/mL isobutyl-methylxanthine (IBMX, Sigma-Aldrich) for 28 days. The formation of intracellular lipid droplets was confirmed by staining cholesterol esters and triacylglycerols with Oil Red O (Merck Chemicals).

Cells were analyzed by use of light microscopy (Olympus, Tokyo, Japan), while differentiation levels were quantified by densitometry, utilizing the NIH ImageJ software (v.1.46r, LOCI; University of Wisconsin, Madison, WI, USA).

### 2.3. CFU–F (Colony Forming Unit–Fibroblast) Assay

To confirm the clonogenic potential of isolated BM-MSCs, their ability to form CFU–F colonies was estimated as described in a previous report [26]. Briefly, cells were plated in triplicate in 24-well plates at a seeding density of 250 cells/well. After 14 days of standard cultivation, cells were washed twice with PBS and fixed using ice-cold methanol. After fixation, cells were stained with 0.3% crystal violet (Carlo Erba reagents S.A.S., Val de Reuil, France) for 15 min, and then washed using distilled water. CFU–Fs that consisted of more than 50 cells and had a diameter lager than 2 mm were observed using light microscopy (Olympus, Tokyo, Japan).

### 2.4. Cellular Viability and Cell Cycle Progression

To evaluate the viability/metabolic activity of BM-MSCs, an MTT test was conducted, as previously reported [27]. BM-MSCs were seeded at 1000 cells per well in 96-well plates and cultivated under standard conditions in GM. After 24 h, cells were treated with different concentrations (0, 10, 20, and 40 nM) of VD3 (cholecalciferol, Santa Cruz Biotechnologies, Dallas, TX, USA) for 24 h, 48 h, 72 h, 5 days, and 7 days, whereby untreated cells served as controls. Afterwards, MTT solution (3-(4,5-dimethylthiazol-2-yl) 2,5-diphenyltetrazolium bromide) (5 mg/mL) (Sigma-Aldrich) was added, and cultures were incubated for another 2 h. The formation of formazan crystals was detected by measuring optical density at 540 nm using an automatic microplate reader (LabSystems Multiskan PLUS, Nelsirrki, Finland).

In separate experiments, when SIRT1 signaling’s involvement in VD3-mediated effects on BM-MSCs was evaluated, cells were treated for 5 days with VD3 (10 or 20 nM) in the presence or absence of the selective SIRT1 inhibitor, 6-chloro-2,3,4,9-tetrahydro-1H-carbazole-1-carboxamide (EX-527, 5 µM) (Santa Cruz Biotechnologies); untreated cells served as controls. After the treatment, an MTT assay was performed as described above.

In addition, the percentage of Ki67-positive cells (as an intracellular proliferation marker) was determined by flow cytometry [26]. For that purpose, cell samples were washed with PBS, fixed in 5% formaldehyde, and permeabilized in 0.5% BSA/PBS containing 0.1% Triton X-100. Following the blocking of nonspecific labeling (30 min in 0.5% BSA/PBS), cells were incubated with rabbit anti-Ki67 antibody (Abcam, Cambridge, UK) for 1 h at room temperature, and afterwards with the secondary anti-rabbit antibody FITC (Sigma-Aldrich) for 30 min at room temperature.

Furthermore, the cells were analyzed for cell cycle progression via flow cytometry, as described previously [28]. After 5-day treatment, BM-MSCs were detached using trypsin/EDTA, and aliquots of 2 × 10^5^ cells were washed with PBS, fixed with absolute ice-cold ethanol, and incubated for 40 min at 37 °C in a PBS solution containing propidium iodide (PI) (Thermo Fisher Scientific, Waltham, MA, USA), 0.1 mg/mL RNase A (Thermo Fisher Scientific), and 0.1% Triton X-100 (Serva Electrophoresis GmBh, Heidelberg, Germany).

### 2.5. Osteogenic and Adipogenic Differentiation of BM-MSCs

To analyze the influence of VD3 on BM-MSCs’ osteogenic and adipogenic differentiation potential, cells were seeded in 96-well plates (5 × 10^3^ cells/well) and cultured in GM under standard conditions until sub-confluence. From that point, cells were cultured in osteogenic (OM) or adipogenic media (AM), in the absence or presence of VD3 (10 nM or 20 nM), for 3 or 4 weeks, respectively, while untreated cells served as controls. In separate experiments studying the influence of 5-day pretreatment with VD3 on BM-MSCs’ osteogenic and adipogenic differentiation potential, as well as the involvement of SIRT1 in these processes, cells were cultured for the first 5 days in GM containing VD3 (10 or 20 nM), with or without EX-527 (5 µM). Afterward, BM-MSCs’ differentiation was induced by culturing BM-MSCs for an appropriate length of time in OM or AM only; untreated cells cultured in GM during the first 5 days were used as controls.

After an appropriate period of differentiation induction, cells were stained and analyzed as described above in Section 2.2.2.

### 2.6. Immunofluorescence Assay

Immunofluorescence staining was performed as previously reported [27]. For that purpose, 3 × 10^3^ cells/well were seeded in 24-well plates over rounded glass coverslips and treated as described in the Results section (Section 3.3, Section 3.4, Section 3.5). Afterwards, cells were fixed in 4% formaldehyde in PBS, permeabilized in 0.1% Triton X-100 in PBS, blocked with 1% BSA/PBS, and incubated at room temperature for 1 h with the following primary antibodies: rabbit anti-Ki67 (Abcam), mouse anti-NANOG, rabbit anti-Oct4, mouse anti-SOX2, rabbit anti-SIRT1, and rabbit anti-FoxO3a (all from Cell Signaling Technology). Subsequently, samples were washed with PBS and incubated with appropriate FITC-coupled secondary antibodies (Sigma-Aldrich) and 1 ng/mL of the nuclear dye DAPI (Sigma-Aldrich) for 2 h. An epifluorescence microscope (Olympus, Tokyo, Japan) was used for the examination of mounted cell samples.

### 2.7. β-Galactosidase Staining

For β-galactosidase staining, BM-MSCs of passages P5 to P7 were seeded at a concentration of 2 × 10^3^ cells/well in 48-well plates and treated as described in the Results section (Section 3.7). After treatment, cells were washed with PBS, fixed, and stained using the Senescence Cells Histochemical Kit according to the manufacturer’s instructions (Sigma-Aldrich, St. Louis, MO, USA). The cells stained for β-galactosidase activity were counted under a light microscope (Olympus, Tokyo, Japan), and the percentage of stained cells was determined for several separated visual fields.

### 2.8. Quantitative Real-Time PCR (qRT-PCR)

BM-MSCs were seeded in 6-well plates at a density of 1 × 10^5^ cells/well and treated as described in the Results section (Section 3.5 and Section 3.6). Afterwards, mRNA was extracted using TRIzol (Ambion Life Technologies, Carlsbad, CA, USA). Concentrations of the samples were measured with a spectrophotometer (Implen, Munich, Germany), and 1 µg of each sample was subjected to reverse transcription using the High-Capacity cDNA Reverse Transcription Kit (Thermo Fisher Scientific). To access relevant genes’ expression, complementary DNA (cDNA) was used as a template for qRT-PCR performed on a Mic qPCR cycler (Bio Molecular Systems, Australia), using primers listed in Table 1 and the Fast Green Kit (Applied Biosystems, Foster City, CA, USA). The relative gene expression was calculated via the comparative ΔΔCt method, with *GAPDH* as a reference [29,30].

### 2.9. Statistical Analysis

All tests were performed three times or more. The results are represented as the average ± SEM. Student’s two-tailed *t*-test was used for evaluation of differences between groups, and *p*-values < 0.05 were considered significant. For data analysis and graphical representations of the performed assays, GraphPad Prism 7 software (GraphPad Software Inc., San Diego, CA, USA) was employed.

## 3. Results

### 3.1. Bone Marrow Mesenchymal Stromal/Stem Cells’ Characterization

**Figure 1:**
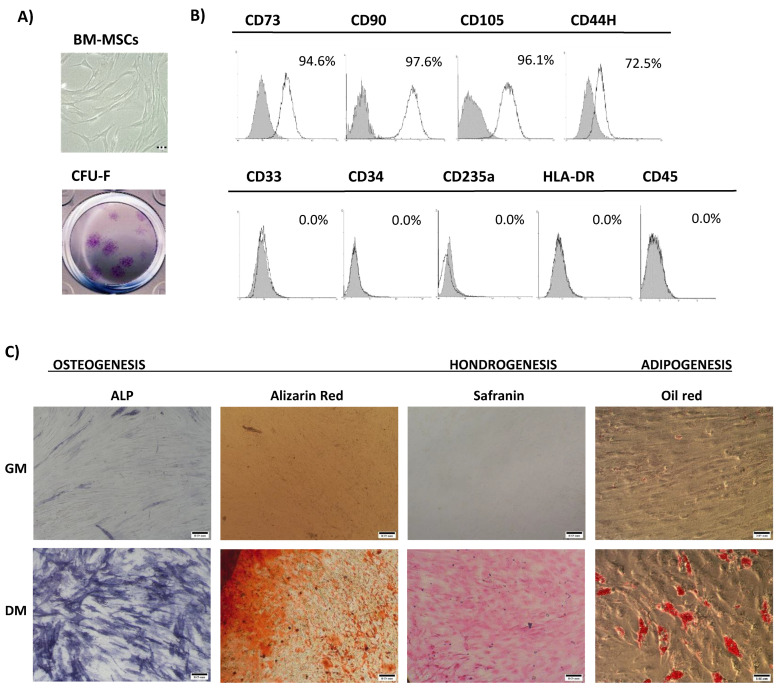
Bone marrow mesenchymal stromal/stem cells’ characterization: (**A**) The upper image shows the fibroblast-like morphology of BM-MSCs; scale bars: 50 μm. The lower image is a representative image of a colony-forming unit–fibroblast (CFU–F) stained with crystal violet. (**B**) The immunophenotype of cells evaluated by flow cytometry. Representative histograms illustrate the percentage of positive cells (empty peaks) relative to isotype controls (shaded peaks). (**C**) Representative images of BM-MSCs’ differentiation potential. Osteogenic differentiation detected at two different time points: after 7 days by positive staining for ALP activity, and after 21 days by positive staining of mineralized deposits by Alizarin Red staining. Scale bars: 50 μm. Chondrogenic differentiation determined by Safranin O staining of proteoglycans after 21 days. Scale bars: 50 μm. Adipogenic differentiation observed after 28 days by staining of intracytoplasmic lipid droplets with Oil Red O staining. Scale bars: 20 μm.

For purpose of BM-MSCs’ characterization, routine analyses of adherence to plastic, immunophenotype, and differentiation potential were conducted in accordance with the minimal criteria proposed by The International Society for Cellular Therapy [24]. Fibroblast-like spindle-shaped morphology of isolated adherent cells was demonstrated, along with their ability to form CFU–F fibroblast-like cell colonies (Figure 1A). Moreover, the immunophenotype evaluation showed positive expression of mesenchymal markers (CD73, CD90, CD105, and CD44H) and an absence of hematopoietic markers (CD33, CD34, CD45, HLA-DR, and CD235a) in isolated and cultivated BM-MSCs (Figure 1B). BM-MSCs’ identity was further confirmed through the detection of their multilineage differentiation capacity. Specifically, after cultivation in the appropriate differentiation-inductive culture conditions, markers of early osteogenesis (ALP), late osteogenesis (mineralization), chondrogenesis (cartilage-specific proteoglycans), and adipogenesis (intracellular lipid droplets) were detected (Figure 1C).

### 3.2. Influence of VD3 on BM-MSCs’ Proliferation and Differentiation

**Figure 2:**
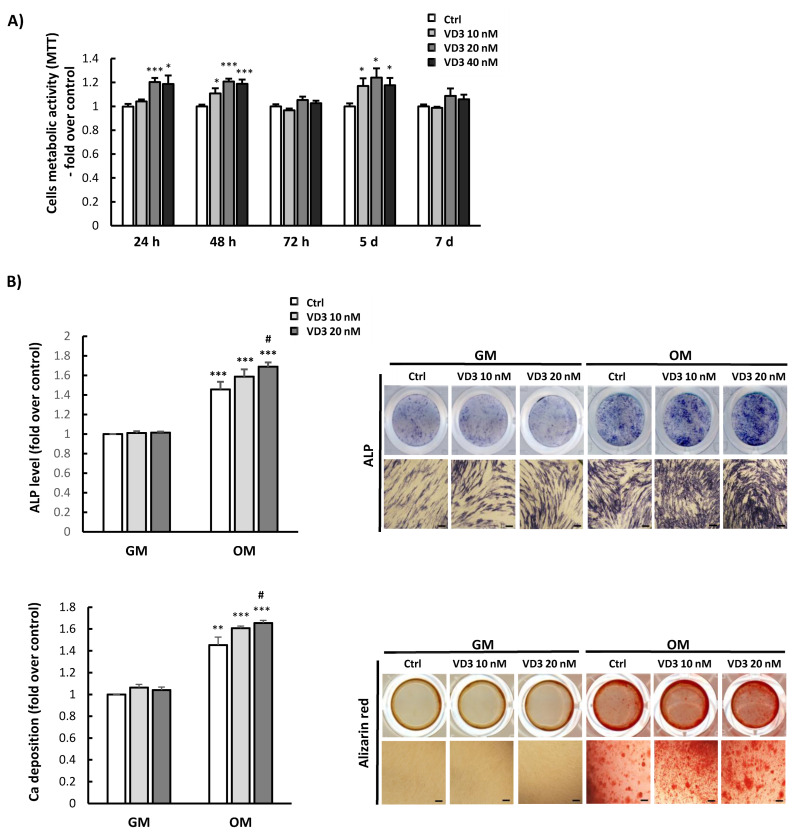
Influence of VD3 on BM-MSCs’ proliferation and differentiation: (**A**) Viability/metabolic activity of cells treated with VD3 (10 nM, 20 nM, or 40 nM) for 24 h, 48 h, 72 h, 5 days, and 7 days, as estimated by MTT assay. Results are presented as the mean ± SEM from at least three independent experiments. Statistically significant differences in comparison to untreated controls (Ctrl): * *p* < 0.05; *** *p* < 0.001. (**B**) Osteogenesis of BM-MSCs was analyzed for cells grown in growth medium (GM) or osteogenic medium (OM), with or without VD3 (10 nM or 20 nM). After 7 days, ALP activity was determined by histochemical staining, while calcium depositions were stained after 21 days with Alizarin Red. Representative images are shown (scale bars: 50 µm). Graphs represent quantitative analysis of ALP activity and mineralization levels for at least three independent experiments. Results are normalized to values for untreated controls and presented as means ± SEM. Statistically significant differences: ** *p* < 0.01; *** *p* < 0.001 compared to untreated GM controls; # *p* < 0.05 compared to untreated OM controls.

Initially, we examined the viability/metabolic activity of BM-MSCs upon treatment with various concentrations of VD3 (10 nM, 20 nM, and 40 nM) for different periods of time. The results of the MTT test indicated that VD3 slightly increased the cell viability and/or metabolic activity of BM-MSCs. Specifically, in comparison to the untreated controls, significantly increased viability of BM-MSCs was detected after 24 h of treatment with 20 nM and 40 nM VD3, while all tested concentrations of VD3 exerted stimulatory effects on the viability/metabolic activity of BM-MSCs after 48 h and 5 days. On the other hand, after 72 h and 7 days of VD3 treatment, no significant differences were observed in cell viability compared to untreated controls (Figure 2A). Since no additional increase in BM-MSCs’ viability was detected at 40 nM, for further investigations, VD3 concentrations of 10 nM and 20 nM were used.

The analyses of VD3′s influence on BM-MSCs’ osteogenic differentiation were next estimated by ALP and Alizarin Red staining. The obtained results, presented in Figure 2B, revealed significant stimulation of early and late osteogenesis of BM-MSCs cultured in osteogenic medium (OM) containing VD3 (10 nM and 20 nM). The highest increase was observed when 20 nM VD3 was added to cultures (Figure 2B). Along with stimulation of osteogenesis, our results showed that VD3 reduced adipogenic differentiation of BM-MSCs in a dose-dependent manner, as the most prominent decrease in the formation of lipid droplets was observed when 20 nM VD3 was present in the adipogenic medium (Figure S1). Moreover, VD3 did not affect the spontaneous differentiation potential of BM-MSCs, given that no changes were detected in ALP activity, mineralization, or intracellular lipid droplet formation during cultivation in GM (Figure 2B and Figure S1). Overall, these results indicate the potential of VD3 to enhance BM-MSCs’ proliferation and osteogenic differentiation, but to inhibit their adipogenic differentiation.

### 3.3. VD3 Activates SIRT1-FoxO3 Signaling Pathways in BM-MSCs

**Figure 3:**
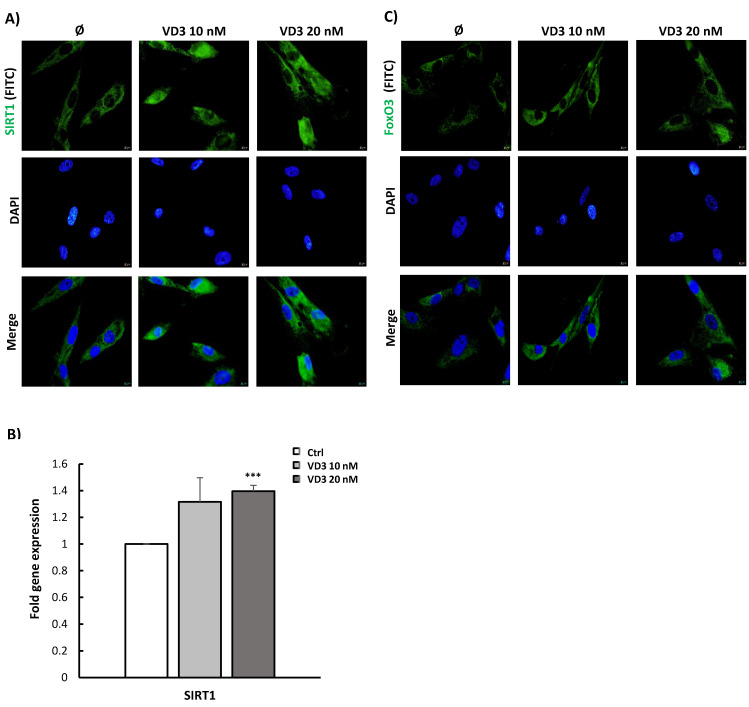
VD3 activates SIRT1-FoxO3 signaling pathways in BM-MSCs: BM-MSCs were treated with VD3 (10 nM or 20 nM) for 5 days under standard cultivation conditions. Protein expression of (**A**) SIRT1 and (**C**) FoxO3 following the treatment, as detected by indirect immunofluorescence staining. Secondary fluorescein isothiocyanate (FITC)-conjugated antibodies were used for labeling corresponding primary antibodies. DNA was stained with 4′,6-diamidino-2-phenylindole (DAPI). Representative images from three independent experiments are shown (scale bars: 10 µm). (**B**) Relative expression levels for SIRT1 mRNA analyzed by qPCR were normalized to the Ct value of the housekeeping gene GAPDH. For calculations, the 2^−ΔΔ^*^CT^* method was used. Results are presented as the mean ± SEM from at least two independent experiments. Statistically significant differences: *** *p* < 0.001 compared to untreated controls.

To evaluate the mechanism underlying VD3-mediated effects on BM-MSCs, we analyzed how VD3 affected SIRT1-FoxO3 signaling pathways in BM-MSCs following the 5-day treatment period. For that purpose, immunofluorescent staining was performed, revealing constitutive protein expression of both SIRT1 and FoxO3—predominantly localized in the cytoplasm of the tested cells. After 5 days of treatment with both concentrations of VD3 (10 nM and 20 nM), our results demonstrated markedly stimulated expression of SIRT1 not only in the cytoplasm of BM-MSCs, but in the nuclear region as well (Figure 3A). Similarly, after 5 days of treatment with VD3 at both concentrations, protein expression of FoxO3 in BM-MSCs was increased—mainly in their cytoplasmic region. Moreover, to a certain extent, treatment with VD3 at 20 nM also increased FoxO3 expression in the perinuclear region of BM-MSCs (Figure 3C). In agreement with the results of immunofluorescent staining, qPCR analysis revealed elevated levels of SIRT1 mRNA expression in BM-MSCs following 5-day incubation with VD3. Statistically significantly higher expression of the *SIRT1* gene was observed at 20 nM VD3 in comparison to untreated controls (Figure 3B).

### 3.4. VD3 Treatment Modulates Proliferation and Cell Cycle Progression of BM-MSCs Independently of SIRT1 Signaling

**Figure 4:**
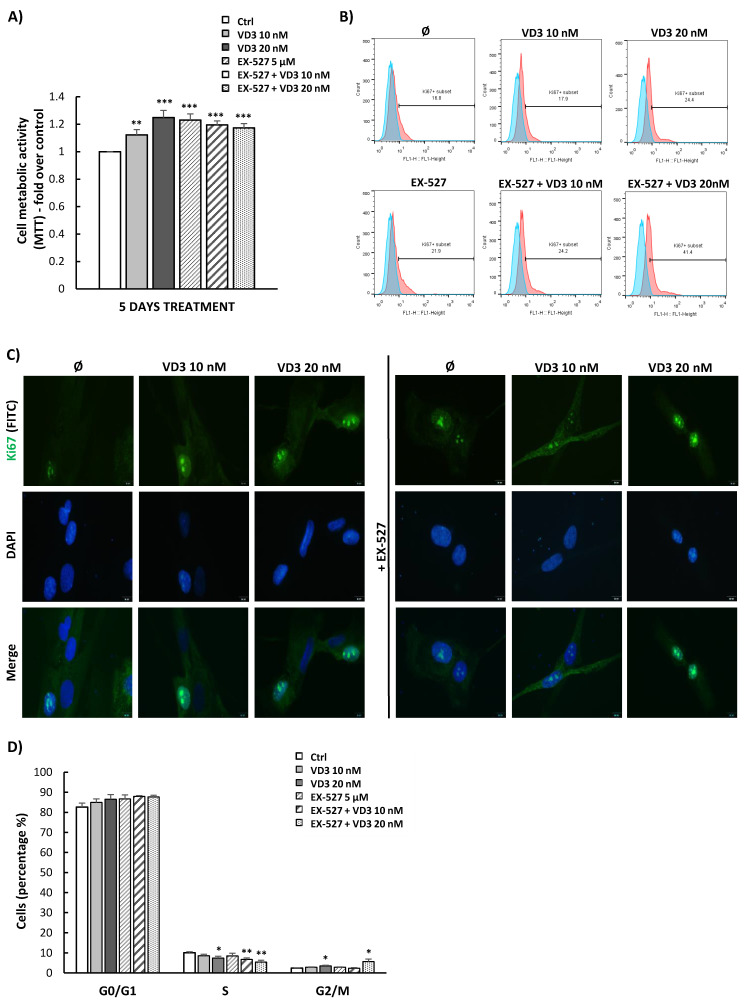
VD3 treatment modulates proliferation and cell cycle progression of BM-MSCs, independently of SIRT1 signaling. BM-MSCs were treated for 5 days with VD3 (10 nM or 20 nM), EX-527 (5 µM), or both, under standard cultivation conditions. (**A**) Metabolic activity estimated by MTT test. Results are presented as the mean ± SEM for at least three independent experiments. Statistically significant differences: ** *p* < 0.01; *** *p* < 0.001 in comparison to untreated controls (Ctrl). (**B**) Expression of the proliferation-associated protein Ki67 in BM-MSCs, as detected by flow cytometry. Representative histograms illustrating the percentage of Ki67-positive cells (pink peaks) versus unstained cells as negative controls (blue peaks). (**C**) Ki67 expression detected by indirect immunofluorescence staining of BM-MSCs. Primary antibodies were labelled with corresponding fluorescein isothiocyanate (FITC)-conjugated secondary antibodies, while DNA was stained with 4′,6-diamidino-2-phenylindole (DAPI). Representative images from three independent experiments conducted in duplicate are shown (scale bars: 10 µm). (**D**) Cell cycle distribution as determined by flow cytometric analysis after propidium iodide (PI) staining. Results from at least three independent experiments are presented in the graph as means ± SEM. Statistically significant differences: * *p* < 0.05; ** *p* < 0.01 in comparison to untreated controls.

As stimulated BM-MSCs’ viability in the presence of VD3 was detected by MTT within a 5-day treatment period, we further intended to estimate the involvement of SIRT1 signaling in this process by using a selective inhibitor of SIRT1: EX-527. After the 5-day treatment, this pharmacological inhibitor successfully reduced the expression of the SIRT1 protein in BM-MSCs at 5 μM concentration (Figure S2) and, at the same time, stimulated the viability of BM-MSCs as determined by MTT (Figure 4A). However, the high metabolic activity of BM-MSCs induced by both EX-527 and VD3 treatment (Figure 4A) indicated that the stimulatory effect of VD3 on BM-MSCs’ viability is SIRT1-independent. In line with the MTT test, results of expression analysis for the proliferation marker Ki67 showed a similar influence of VD3 and EX-527. Specifically, the 5-day treatment of BM-MSCs with VD3 increased the percentage of Ki67-positive cells—particularly in the presence of higher VD3 concentrations (Figure 4B). However, an increased proportion of Ki67-positive cells was also observed upon EX-527 treatment, and it was additionally stimulated when applied together with 20 nM VD3 (Figure 4B). Immunofluorescence analyses of Ki67 expression demonstrated increased nuclear expression of this marker in VD3-treated BM-MSCs (Figure 4C), whereby the presence of EX-527 did not change this effect (Figure 4C). Moreover, analysis of cell cycle progression revealed that the treatment with 20 nM VD3 slightly reduced the percentage of BM-MSCs in the S phase, while in parallel it increased their percentage in the G2/M phase (Figure 4D). Again, this effect was not changed in the presence of EX-527. Collectively, these findings indicate that VD3 can influence BM-MSC proliferation, and that this effect does not depend on the activation of SIRT1 signaling in these cells.

### 3.5. VD3 Treatment Modulates the Expression of Pluripotency-Associated Markers in BM-MSCs: Involvement of SIRT1 Signaling

**Figure 5:**
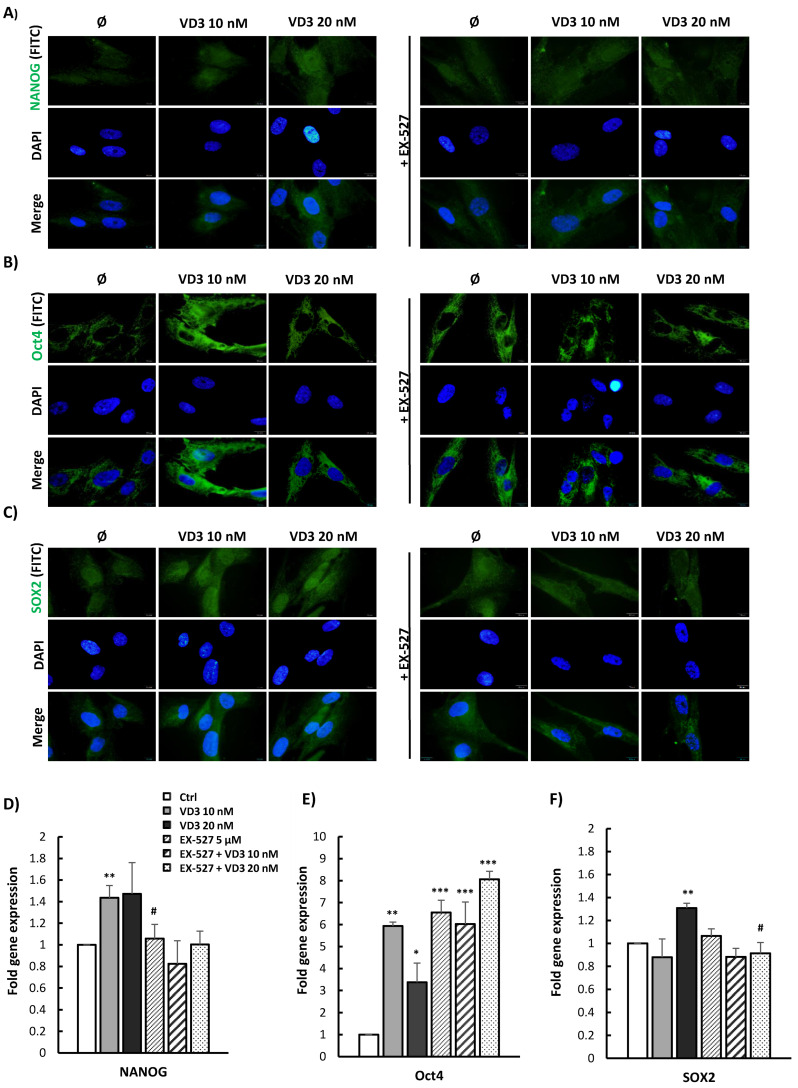
VD3 treatment modulates the expression of pluripotency-associated markers in BM-MSCs: involvement of SIRT1 signaling: BM-MSCs were treated for 5 days with VD3 (10 nM or 20 nM), EX-527 (5 µM), or both. Immunocytochemical detection of pluripotency-related transcription factors: (**A**) NANOG, (**B**) Oct4, and (**C**) SOX2. Primary antibodies were labeled with appropriate FITC-conjugated secondary antibodies, while cell nuclei were stained with 4′,6-diamidino-2-phenylindole (DAPI). Representative images from three independent experiments conducted in triplicate are shown (scale bars: 10 µm). Graphical presentation of pluripotency markers’ mRNA expression as analyzed by qPCR: relative gene expression levels for (**D**) NANOG, (**E**) Oct4, and (**F**) SOX2, normalized to the Ct value of the housekeeping gene GAPDH. Calculations were performed by applying the 2^−ΔΔ^*^CT^* method, and results were presented as the mean ± SEM from at least two independent experiments. Statistically significant differences: * *p* < 0.05; ** *p* < 0.01; *** *p* < 0.001 compared to untreated controls; # *p* < 0.05 compared to corresponding VD3 treatment without EX-527.

To further estimate the influence of VD3 treatment on stemness features of BM-MSCs, and the potential involvement of SIRT signaling in this process, the expression of pluripotency-associated markers was examined following the 5-day treatment with VD3 (10 nM or 20 nM), in the presence or absence of EX-527 (5 µM). Expression of the transcription factors NANOG, Oct4, and SOX2—as key players in the regulation of self-renewal and pluripotency of stem cells—was analyzed at the protein and gene levels. The results of fluorescent immunostaining presented in Figure 5 showed constitutive protein expression of all three analyzed transcription factors in untreated BM-MSCs. While weak positive staining was detected for NANOG and SOX2 in both the cytoplasmic and nuclear regions of untreated cells, Oct4 staining was evidenced only within their cytoplasm. Moreover, the 5-day treatment with VD3 stimulated protein expression of NANOG, Oct4, and SOX2 in BM-MSCs, although some differences were noted in the expression patterns of the tested markers. Elevated expression of NANOG induced by VD3 was observable in both cell compartments, and was clearly attenuated in the presence of EX-527 (Figure 5A). Similarly, BM-MSCs showed increased SOX2 expression in the cytoplasm and nucleus after 5-day treatment with both concentrations of VD3, while treatment with EX-527 reduced the stimulatory effect of VD3 on SOX2 expression—particularly in the nuclear region (Figure 5C). Furthermore, upon treatment with VD3 at a concentration of 10 nM, marked stimulation of Oct4 expression was observed in BM-MSCs’ cytoplasm (Figure 5B). On the other hand, the 5-day treatment with 20 nM VD3 led to a less pronounced increase in Oct4 expression than with 10 nM VD3. Treatment with EX-527 alone or combined with VD3 also considerably stimulated the expression of Oct4 in the cytoplasmic region of BM-MSCs. Likewise, the results of qPCR analyses evidenced higher expression levels of the mRNA of the pluripotency markers NANOG, Oct4, and SOX2 in BM-MSCs treated for 5 days with VD3 (Figure 5D–F). In addition, while EX-527 significantly attenuated the positive influence of VD3 on NANOG and SOX2 mRNA expression, this inhibitor did not modify the effect of VD3 on Oct4 mRNA expression—on the contrary, higher levels of Oct4 mRNA expression were detected in BM-MSCs treated with EX-527, either alone or combined with VD3 (Figure 5E). Taken together, our obtained findings demonstrate a stimulatory effect of VD3 on the expression of pluripotency-associated markers in BM-MSCs, suggesting potential involvement of the SIRT1 signaling pathway in VD3-modulated NANOG and SOX2 expression, but not in the expression of Oct4.

### 3.6. Modulated Differentiation Potential of VD3-Pretreated BM-MSCs: Involvement of SIRT1 Signaling

**Figure 6:**
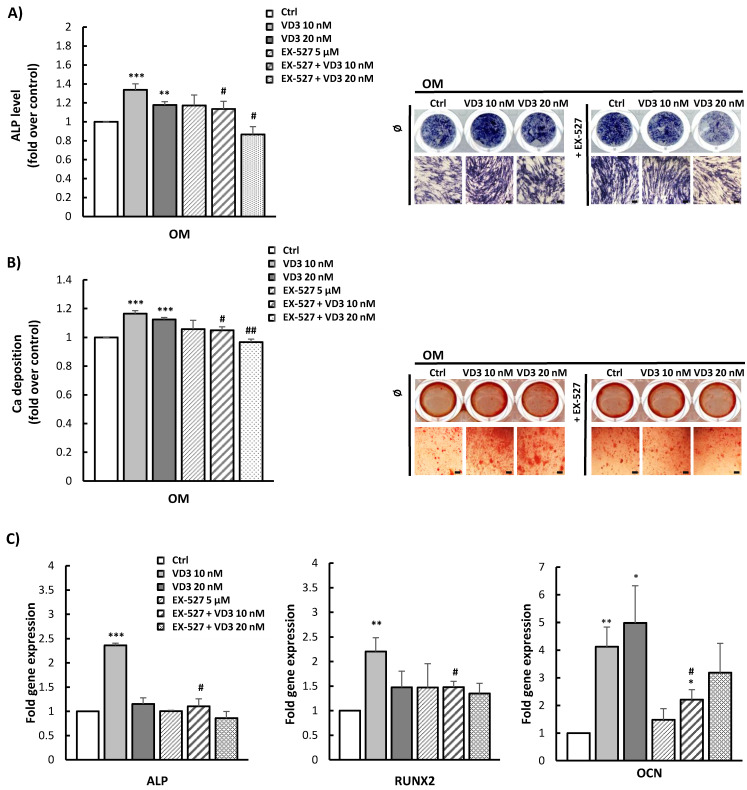
Stimulated osteogenic differentiation of VD3-pretreated BM-MSCs: involvement of SIRT1 signaling: BM-MSCs were pretreated for 5 days with VD3 (10 nM or 20 nM), EX-527 (5 µM), or both, in GM. Afterwards, the cells were cultured in OM for the appropriate period to induce osteogenesis. (**A**) ALP activity detected after 7 days of cultivation in OM by histochemical staining. (**B**) Calcium depositions stained with Alizarin Red after 21 days. Representative images are shown (scale bars: 50 µm). Graphs represent quantitative analysis of (**A**) ALP activity and (B) mineralization level for at least three independent experiments. Results are normalized to values for untreated controls and presented as means ± SEM. Statistically significant differences: ** *p* < 0.01; *** *p* < 0.001 compared to untreated OM controls; # *p* < 0.05, ## *p* < 0.01 compared to corresponding pretreatment with VD3 in the absence of EX-527. (**C**) For quantitative real-time PCR analysis of the ALP, RUNX2, and OCN mRNA expression, cells were grown for 5 days in GM in the presence of VD3 (10 nM or 20 nM), EX-527 (5 µM), or both. Relative expression levels for ALP, RUNX2, and OCN genes were normalized to the Ct value of the housekeeping gene GAPDH. Calculations by the 2^−ΔΔ^*^CT^* method are presented as the mean ± SEM from at least two independent experiments. Statistically significant differences: * *p* < 0.05; ** *p* < 0.01; *** *p* < 0.001 compared to untreated controls; # *p* < 0.05 compared to corresponding VD3 treatment without EX-527.

To investigate the potential of VD3 treatment to enhance the regenerative features of BM-MSCs—particularly in terms of their osteogenic differentiation capacity—as well as the role of SIRT1 signaling in VD3-mediated effects, BM-MSCs were pretreated for 5 days with VD3 (10 nM or 20 nM) in the presence or absence of EX-527 (5 µM), and subsequently induced to osteogenesis. Obtained results showed a significant potential of VD3 to stimulate osteogenic differentiation of BM-MSCs after 5-day pretreatment at both concentrations applied. This potential of VD3 was observed for both early and late osteogenesis, as detected by ALP and Alizarin Red staining, respectively (Figure 6A,B). On the other hand, the application of EX-527 alone did not modify the osteogenic potential of BM-MSCs, while it blocked the stimulative effects of VD3 on both ALP expression and mineralization. This blocking effect was the most pronounced when EX-527 was combined with 20 nM VD3 (Figure 6A,B). Gene expression analysis of osteogenesis-related markers, as assessed by qPCR after the 5-day treatment with VD3 in the presence or absence of EX-527, confirmed the observed osteoinductive effects of VD3. While statistically significant stimulation of ALP and RUNX2 gene expression was detected in the presence of 10 nM VD3, both tested concentrations of VD3 stimulated OCN gene expression (Figure 6C). However, EX-527 almost completely diminished upregulated gene expression of all tested osteogenesis-related markers (ALP, RUNX2, and OCN) (Figure 6C). Despite that, when the influence of 5-day treatment with VD3—in the presence or absence of EX-527—on subsequent adipogenesis of BM-MSCs was analyzed, a significant potential of this vitamin to reduce adipogenic differentiation of pretreated cells was demonstrated. In this case, treatment with EX-527 did not significantly modify VD3-inhibited lipid droplet formation in BM-MSCs (Figure S3).

Overall, the collected results indicate that VD3 treatment, even at lower concentrations, can enforce the osteogenic differentiation potential of BM-MSCs, while the reducing effect of EX-527 points to the involvement of SIRT1 signaling in this process. On the other hand, the observed inhibitory effect of VD3 pretreatment on BM-MSCs’ adipogenesis seems not to require SIRT1 to mediate this phenomenon.

### 3.7. VD3 Treatment Reduces β-Galactosidase Expression in BM-MSCs: The Role of SIRT1 Signaling

**Figure 7:**
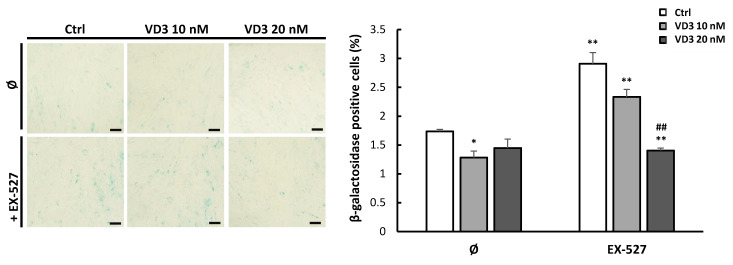
VD3 treatment reduces β-galactosidase expression in BM-MSCs: the role of SIRT1 signaling: Identification of β-galactosidase-positive cells was conducted following 5-day treatment with VD3 (10 nM or 20 nM), EX-527 (5 μM), or both, in GM. Representative images of β-galactosidase-positive cells (**left**); scale bars: 50 μm. The percentage of β-galactosidase-positive cells was enumerated per visual field and presented as the mean ± SEM of three independent experiments (**right**). Statistically significant differences: * *p* < 0.05, ** *p* < 0.01 compared to untreated controls (Ø); ## *p* < 0.01 compared to corresponding VD3 treatment without EX-527.

Additional experiments were conducted with the aim to evaluate the impact of VD3 treatment on cellular aging (senescence) of BM-MSCs, as well as the potential involvement of SIRT1 in VD3-mediated effects. For this purpose, the expression of the senescence marker β-galactosidase was analyzed in BM-MSCs after 5-day treatment with VD3 (10 nM or 20 nM), EX-527 (5 µM) or both. Results demonstrated a lower percentage of β-galactosidase-positive cells in VD3-treated BM-MSCs, reaching statistical significance when 10 nM VD3 was applied (Figure 7). As expected, inhibition of SIRT1 by EX-527 resulted in an increased level of β-galactosidase-positive cells, in accordance with the protective role of SIRT1 in cellular senescence (Figure 7). However, when combined with VD3, the senescence-promoting activity of EX-527 on BM-MSCs was attenuated, as demonstrated by a lower proportion of β-galactosidase-expressing cells—particularly when 20 nM VD3 was used. These results imply that VD3 treatment might potentially delay the replicative senescence of BM-MSCs, independently of SIRT1 signaling.

## 4. Discussion

The biological potential of cholecalciferol (VD3) within regenerative medicine has not been sufficiently investigated. Recent studies have implied its significance in tissue engineering, showing that its incorporation in various biomaterial matrices or 3D scaffolds may improve MSCs’ regenerative potential [7,8,9]. In addition, preconditioning/priming procedures represent some of the key strategies to improve MSCs’ function and enable more direct translation to clinical practice. However, the use of VD3 as a pretreatment factor in BM-MSCs’ transplantation protocols has not been investigated thus far. Therefore, this study aimed to evaluate the effects of VD3 treatment on functional features of BM-MSCs, as well as the potential involvement of the SIRT1 signaling pathway in these effects. For this purpose, BM-MSCs’ proliferation capacity and expression of pluripotency- and senescence-associated markers, along with their differentiation potential, were analyzed following a 5-day treatment with VD3.

In general, data related to the impact of vitamin D3 on MSC proliferation are variable, depending on the tissue source and species, as well as the concentration and the form in which it is applied. Particularly, depending on the cell type, vitamin D3 has been shown to exert both stimulatory and inhibitory effects on cell proliferation by altering different processes, including cell cycle progression, apoptosis, and senescence [31,32]. Specifically, the stimulatory effect of the VD3 metabolite 1α,25(OH)_2_D3 on rat BM-MSCs’ growth was already visible after 1-day incubation at 100 nM and 1 μM concentrations, as well as after 3 days at a lower concentration (10 nM) [33,34]. In addition, increased proliferation of human and murine BM-MSCs as a result of 1α,25(OH)_2_D3 treatment was also demonstrated, along with the reduction in their senescence [35,36]. However, few authors have studied the effects of vitamin D3 in its native form (VD3, cholecalciferol) instead of its more active metabolites. Chen et al. designed and fabricated a composite cement containing calcium citrate and calcium sulfate with incorporated VD3 that is slowly, sustainably, and controllably released from the composite in a quantity-dependent manner, which showed a stimulatory effect on murine BM-MSCs’ viability and proliferation rate [7]. Moreover, when the effects of VD3 and its metabolites in different concentrations on the functional features of osteoblasts from neonatal rat calvariae were investigated, only 200 nM VD3 significantly promoted osteoblast proliferation, while smaller concentrations of VD3 and the metabolites did not exert significant effects [37]. The results presented in our study demonstrate that VD3 increased proliferation of human BM-MSCs obtained from young donors after 1 day at higher concentrations (20 nM and 40 nM), while this effect was noticed after longer 2-day and 5-day incubation periods at all tested concentrations. The most noticeable effect was observed at 20 nM concentration of VD3 after 5 days, as evidenced by increased metabolic activity and Ki67 expression in treated BM-MSCs.

On the other hand, antiproliferative effects of the vitamin D3 metabolite 1α,25(OH)_2_D3 have been shown in C3H 10T1/2 murine multipotent mesenchymal cells after 4-day treatment at concentrations ranging from 25 to 100 nM [38]. Similar results have been reported for human BM-MSCs, on which 1α,25(OH)_2_D3 exerted an antiproliferative effect at higher concentrations after 3 days, as well as at lower concentrations after a longer, 5-day incubation period [39,40]. These differences in vitamin-D3-modulated effects on MSC proliferation may be attributed to different tissue sources, donors’ age, and different concentrations and/or different forms of vitamin D3 used. It has been shown that human MSCs can express all hydroxylases involved in VD3 metabolism (CYP2R1, CYP27A1, CYP27B1, and CYP24A1) that are needed for its conversion to the active form [13]; however, not all MSCs express these enzymes equally. In human BM-MSCs with high constitutive expression of CYP27B1 (1α-hydroxylase), both 25(OH)D3 and 1α,25(OH)_2_D3 decreased their proliferation in a dose-dependent manner, while those BM-MSCs with low levels of this enzyme were resistant to 25(OH)D3 [15]. Another specificity of our study is that the donors were of young age, and most other studies carried out on human BM-MSCs have been from hip arthroplasty patients, with diverse clinical attributes that could influence the proliferation capacity of MSCs and their responsiveness to vitamin D3 [41].

During the past decade, many studies have indicated an important role of SIRT1 signaling in MSC self-renewal, demonstrating its protective effects against DNA damage, reactive oxygen species, and various inflammatory and apoptotic stimuli [22]. Moreover, SIRT1 has been implicated in the promotion of murine and human BM-MSCs’ proliferation, and its role in bone homeostasis is generally well documented [37,42,43,44]. It has been also shown that vitamin D3 exerts its effects on MSCs by increasing SIRT1 expression via VDR-mediated transcription [43,45]. Accordingly, our results confirmed the ability of VD3 to stimulate the expression of SIRT1, as well as its downstream target FoxO3, in BM-MSCs. Although in most cell types SIRT1 localizes in the nucleus, it can also be found in the cytoplasm and subjected to nucleocytoplasmic shuttling [46]. In fact, we found constitutive expression of SIRT1 in the BM-MSCs’ cytoplasm, but also in their nucleus after VD3 treatment. Nevertheless, further studies are needed in order to elucidate the role of SIRT1 localization in the regulation of BM-MSCs’ functional features. However, as for the BM-MSCs’ proliferation, our study showed that the addition of the SIRT1 inhibitor EX-527 did not block the stimulatory effect of VD3 on BM-MSC proliferation, suggesting that this effect of VD3 is not mediated via SIRT1.

Pluripotency-associated transcription factors—including NANOG, Oct4, and SOX2—play an essential role in maintaining the stemness of embryonic stem cells [47,48], but have also been related to stemness features of MSCs. Although previous studies have shown that pluripotency markers can also act as regulators of self-renewal and the multilineage capacity of adult stem cells [49,50], the significance of NANOG, Oct4, and SOX2 expression in BM-MSCs has not been completely elucidated. Our study evidenced constitutive expression of all examined markers—NANOG, Oct4, and SOX2—in human BM-MSCs, consistent with previous findings of other groups that demonstrated their presence in various types of MSCs [51,52,53]. Moreover, we showed the ability of VD3 to stimulate the expression of NANOG, Oct4, and SOX2 at the protein and gene levels in these cells. Similarly, few studies have also shown a stimulatory effect of 1α,25(OH)2D3 on NANOG expression in human BM-MSCs [39], or on Oct4 expression at the gene level in murine BM-MSCs [54]. On the other hand, no significant differences in NANOG, SOX2, and Oct4 expression at the transcript level were found in the adipose tissue MSCs after treatment with vitamin D3 [17], indicating that this vitamin may differently affect the expression of pluripotency-associated factors in a tissue-source-dependent manner. A positive correlation between cell proliferation and levels of NANOG and Oct4 expression has been demonstrated in human BM-MSCs overexpressing these pluripotent markers [55]. Furthermore, forced expression of SOX2 and NANOG in human BM-MSCs induced by retroviral infection demonstrated that tested transcription factors affect the expansion and the differentiation potential of these MSCs [56]. According to Piccinato et al., a low expression of p16INK4A protein and a high *Oct4* gene expression may be potential predictors of an extended in vitro life span and the growth potential of BM-MSCs [57]. Whether elevated expression of NANOG, Oct4, and SOX2 after VD3 treatment in BM-MSCs can be correlated with the increased proliferation and metabolic activity observed in this study needs to be further elucidated.

A few studies have indicated the involvement of SIRT1 signaling in the regulation of the expression of pluripotency-associated markers in stem cells. Specifically, it has been reported that treatment with the SIRT1 activator resveratrol improved the stability of SOX2 in human BM-MSCs [58], while a selective inhibitor of SIRT1—EX-527—downregulated expression of NANOG, SOX2, and Oct4 at the gene level in P19 murine pluripotent cells [59]. However, our results did not reveal decreased expression of the examined markers following treatment with EX-527 alone. Moreover, EX-527 diminished the positive effect of VD3 on NANOG and SOX2 expression at both the protein and gene levels, indicating the role of SIRT1 in VD3-mediated stimulation of these factors. On the other hand, Oct4 expression appeared not to be attenuated by EX-527. Indeed, EX-527—either alone or combined with VD3—elevated Oct4 expression in BM-MSCs, most notably at the mRNA level. Similar to our results, Wang et al. demonstrated that inhibition of SIRT1 by trichostatin and vorinostat in goat adipose tissue MSCs led to hyperacetylation of histone H3 in lysine 9 (H3K9), which promoted Oct4 gene transcription without significantly affecting the protein expression [60]. The same study also demonstrated downregulation of SOX2 expression at both the mRNA and protein levels, but the promotion of NANOG expression upon SIRT1 inhibition.

Vitamin D also plays a significant role in the regulation of the aging process—primarily through anti-aging activities that, consequently, contribute to the prevention of various age-related diseases [61,62,63]. At the cellular level, 1α,25(OH)_2_D3 has been found to exert anti-aging effects via inhibition of oxidative stress and DNA damage, downregulation of p16/Rb and p53/p21 signaling, and reduction in cellular senescence and senescence-associated secretory phenotype (SASP) [64]. As for BM-MSCs, it has been shown that 100 nM 1α,25(OH)_2_D3 significantly prevents replicative senescence during long-term cultivation [39], while recent results have also demonstrated that this metabolite inhibits BM-MSC senescence through VDR-mediated transcriptional upregulation of Ezh2 and inhibition of p16/p19 signaling [36]. The results of our study are consistent with these data, given that we observed a significant decrease in the numbers of senescent BM-MSCs after a 5-day VD3 treatment. Moreover, our results also showed that SIRT1 inhibition stimulated BM-MSCs’ cellular senescence, in agreement with previous studies evidencing SIRT1′s contribution to the prevention of MSCs’ senescence [42,65,66,67]. However, our results did not confirm the findings showing that vitamin-D3-mediated inhibition of senescence involves SIRT1 activation as it does in endothelial cells [68,69] and human-mandible-derived BM-MSCs [43]. Specifically, even in the presence of EX-527, VD3 led to the significant inhibition of BM-MSC senescence, indicating that VD3 exerted its anti-senescence effects in BM-MSCs via activation of other signaling pathways, which should be clarified in future studies. Although some studies point to the correlation between increased expression of pluripotency markers and delayed senescence in MSCs [57,70], further studies are needed in order to confirm this correlation under our experimental conditions.

When it comes to the literature data on the influence of vitamin D3 on osteogenic differentiation, stimulatory effects have been reported. Most of the studies were conducted with vitamin D3 metabolites, while very few data on native VD3 impact are available. Specifically, it has been found that VD3 did not modulate the expression of osteogenic markers in osteoblasts isolated from rat calvariae; however, when these cells were treated with its metabolites—25(OH)D3 and 1α,25(OH)_2_ D3—increased expression of the early osteogenic markers ALP and RUNX2 was shown, while only 25(OH)D3 increased the mineralization level in parallel with OCN expression [19]. In addition, when human osteoblasts were pretreated for 2 days with 50 nM 1α,25(OH)_2_D3, a 14-fold increase in OCN gene expression was detected [71]. Moreover, according to the evidence that human MSCs are not only responsive to 1α,25(OH)_2_D3, but also have the ability to produce it, a potential autocrine/paracrine role of vitamin D3 metabolism in osteogenesis has been hypothesized [13]. Specifically, Geng et al. evidenced that human MSCs express all components of VD3-metabolizing machinery, and that MSCs’ osteogenesis in vitro can be stimulated by 25(OH)D3 in a 1α-hydroxylase-dependent manner [13,15]. A stimulatory effect of 1α,25(OH)_2_D3 on osteogenic differentiation of BM-MSCs grown in 3D spheroids has also been demonstrated, showing significantly stimulated ALP activity even at a low concentration (0.1 nM) of this metabolite [72]. In addition, several studies utilizing various biomaterial scaffolds or composites loaded with VD3 have demonstrated its osteoinductive effects on MSCs [6,20,73]. Specifically, it was found that VD3 in combination with nano-HAP loaded on polycaprolactone/gelatin scaffolds stimulated osteogenesis in adipose tissue MSCs, as evidenced by increased ALP activity and mineralization, as well as gene expression of osteogenic markers (e.g., COLL I, ALP, RUNX2, BGLAP) [6]. Similarly, VD3 loaded on cellulose-enriched HAP/silica scaffolds induced increased osteogenesis of an osteoblast-like cell line (MG63), as evidenced by elevated expression of the ALP, RUNX2, and OCN genes [21], while VD3/vitamin K2/Mg-loaded polymers’ composite nanofibers supported osteogenic differentiation of BM-MSCs, increasing the expression of RUNX2, BMP2, and osteopontin [20]. In addition, human dental pulp MSCs grown on chitosan scaffolds constructed to sustainably deliver VD3 over the course of 3–5 days showed higher expression of ALP, RUNX2, and OCN, even when cultured in standard growth medium [73]. Comparable to these findings, our results demonstrated stimulatory effects of both treatment and pretreatment with VD3 on BM-MSCs’ osteogenesis, as evidenced by enhanced ALP activity and mineralization, along with increased expression of osteogenesis-related genes (ALP, RUNX2, and OCN). Our obtained data suggest that VD3 in the form of cholecalciferol can promote osteogenesis of BM-MSCs from young donors better than its metabolites examined in the studies of other groups. As for the involvement of SIRT1 in the regulation of osteogenic differentiation, a key role of this signaling pathway has been well documented [44,74], as has its potential to enhance the pro-osteogenic effect of the VD3 metabolite 1α,25(OH)_2_D3 through deacetylation of VDR and epigenetic modulation of its target genes [75]. In line with these data, our results showed that SIRT1 inhibition by EX-527 attenuated the positive effect of VD3 on osteogenic differentiation, suggesting that VD3 exerted its influence on BM-MSCs’ osteogenesis at least in part through SIRT1 signaling.

Osteogenic and adipogenic differentiation in BM-MSCs are counter-regulated, and are usually in equilibrium [76]. Consequently, as vitamin D3 apparently promotes osteogenic differentiation of BM-MSCs, it is not unexpected that our results showed decreased adipogenic differentiation upon VD3 treatment. Indeed, 1,25(OH)_2_ D3 has been known to inhibit adipogenesis of murine preadipocytes and BM-MSCs [77,78]. However, this inhibitory effect of vitamin D3 is not straightforward, and some authors have reported a dose-dependent modulation of adipogenesis by 1,25(OH)_2_D3, whereby lower concentrations (0.1 nM) suppressed the expression of adipogenesis markers, while higher (10 nM) concentrations enhanced it [79]. SIRT1 has been shown to attenuate the development of adipocytes from preadipocytes [80], and to inhibit PPAR-γ2 expression in mesenchymal C3H10T1/2 cells and primary rat bone marrow stromal cells [81]. Our results, on the other hand, show that SIRT1 plays no considerable role in mediating the effects of VD3 on adipogenesis, suggesting that other mechanisms may also be involved.

## 5. Conclusions

In conclusion, the results of this study provide new evidence related to the potential of vitamin D3/cholecalciferol (VD3) to improve the regenerative potential of BM-MSCs, and offer a possible mechanistic basis of its action, particularly supporting its use as a supplement in bone regeneration strategies. Specifically, our results demonstrate that treatment with VD3 increases viability/proliferation, expression of pluripotency-associated markers (i.e., NANOG, SOX2, and Oct4), and osteogenic differentiation of BM-MSCs, while also reducing their senescence and adipogenic differentiation potential. At the molecular level, VD3 stimulated expression of SIRT1 in BM-MSCs, whose involvement in VD3-stimulated osteogenesis—as well as in NANOG and SOX2 upregulation—was further revealed. In contrast, the effects of VD3 on proliferation, Oct4 expression, senescence, and adipogenesis were found to be SIRT1-independent. These data indicate that human BM-MSCs from young donors are also responsive to VD3 in its native form, confirming that BM-MSCs possess molecular machinery for metabolizing VD3. Thus, these findings support the use of VD3 in bio-scaffolds, but also open up new possibilities for its potential use as a pretreatment factor in BM-MSC transplantation protocols. However, further studies of VDR, 25-hydroxylase, 1α-hydroxylase, and 24-hydroxylase expression in these cells need to be performed to discover the underlying mechanism involved in VD3 metabolism, in order to contribute to the development of clinically acceptable protocols in cell therapy and tissue engineering.

## Figures and Tables

**Table 1:** PCR primer sets used in experiments.

Gene	Sequence (5′-3′)
*GAPDH*	F: GAAGGTGAAGGTCGGAGT
	R: GAAGATGGTGATGGGATTTC
*ALP*	F: CACCCACGTCGATTGCATCT
	R: TAGCCACGTTGGTGTTGAGC
*OCN*	F: GGCGCTACCTGTATCAATGG
	R: TCAGCCAACTCGTCACAGTC
*RUNX2*	F: GCCTAGGCGCATTTCAGA
	R: CTGAGAGTGGAAGGCCAGAG
*NANOG*	F: GATGCCTCACACGGAGACTG
	R: GCAGAAGTGGGTTGTTTGCC
*Oct4*	F: TGAGTAGTCCCTTCGCAAGC
	R. GCTTCGGATTTCGCCTTCTC
*SOX2*	F: GACAGTTACGCGCACATGAA
	R: TAGGTCTGCGAGCTGGTCAT
*SIRT1*	F: TGCTGGCCTAATAGAGTGGCA
	R: CTCAGCGCCATGGAAAATGT

## Data Availability

The data presented in this study are available from the corresponding author upon reasonable request.

## Supplementary Materials

The following supporting information can be downloaded at: https://www.mdpi.com/article/10.3390/biom12020323/s1, Figure S1: VD3 modulates adipogenic differentiation of BM-MSCs, Figure S2: EX-527 inhibits SIRT1 expression in BM-MSCs, Figure S3: Reduced adipogenic differentiation of VD3-pretreated BM-MSCs: role of SIRT1 signaling.
