# Supplementary material for: Vitamin D3 Stimulates Proliferation Capacity, Expression of Pluripotency Markers, and Osteogenesis of Human Bone Marrow Mesenchymal Stromal/Stem Cells, Partly through SIRT1 Signaling
Source: Biomolecules. 2022 Feb 18;12(2):323. doi: 10.3390/biom12020323 (PMC8868595; doi:10.3390/biom12020323)
Supplement: Supplementary file 1 [file biomolecules-12-00323-s001.zip › SUPPLEMENTARY FIGURES.pdf]

SUPPLEMENTARY FIGURES AND FIGURE LEGENDS

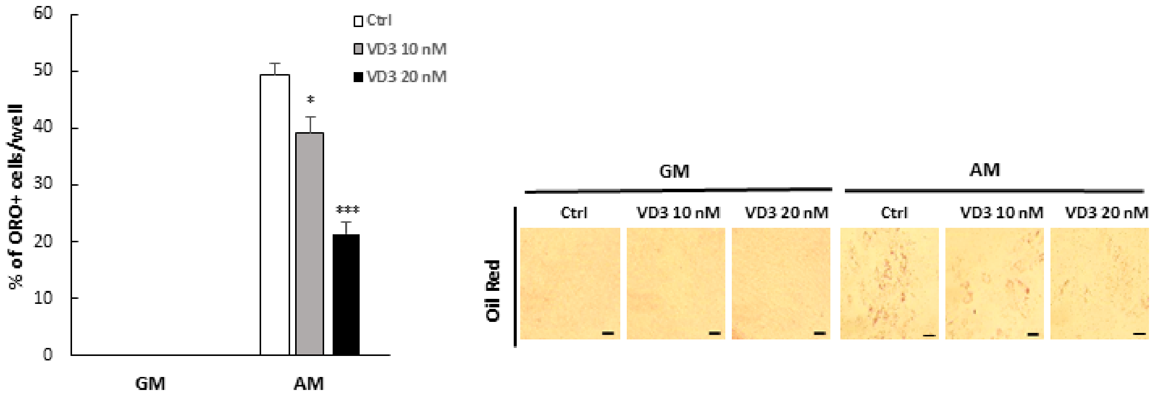

Figure S1. VD3 modulates adipogenic differentiation of BM-MSCs. Adipogenesis of BM-MSCs was analyzed for cells grown in GM or adipogenic medium (AM) with or without VD3 (10nM or 20nM). After 28 days intracellular lipid droplets were detected by Oil Red O staining. Representative images are shown (Scale bars: 20μm). Graph illustrates quantitative analysis showing Oil Red O positive (ORO+) cells percentage enumerated per visual field. Results are presented as means ± SEM for at least three independent experiments. Statistically significant differences by t test: *$p < 0.05$; ***$p < 0.001$ compared to untreated AM control.

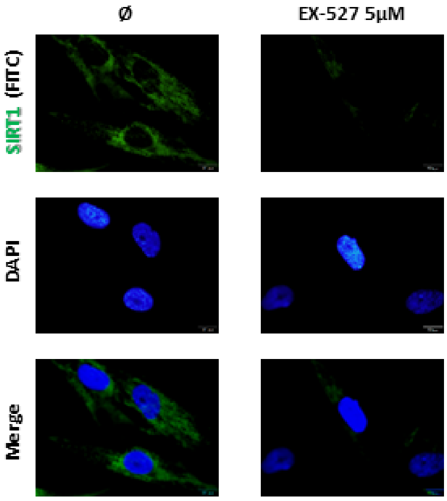

Figure S2. EX-527 inhibits SIRT1 expression in BM-MSCs. BM-MSCs were treated with or without EX-527 (5µM) during 5 days under standard cultivation conditions in GM. Protein expression of SIRT1 following the treatment detected by indirect immunofluorescence staining. Secondary fluorescein isothiocyanate (FITC)-conjugated antibodies were used for labeling corresponding primary antibodies. DNA was stained with 4′,6-diamidino-2-phenylindole (DAPI). Representative images from two independent experiments are shown (Scale bars: 20µM).

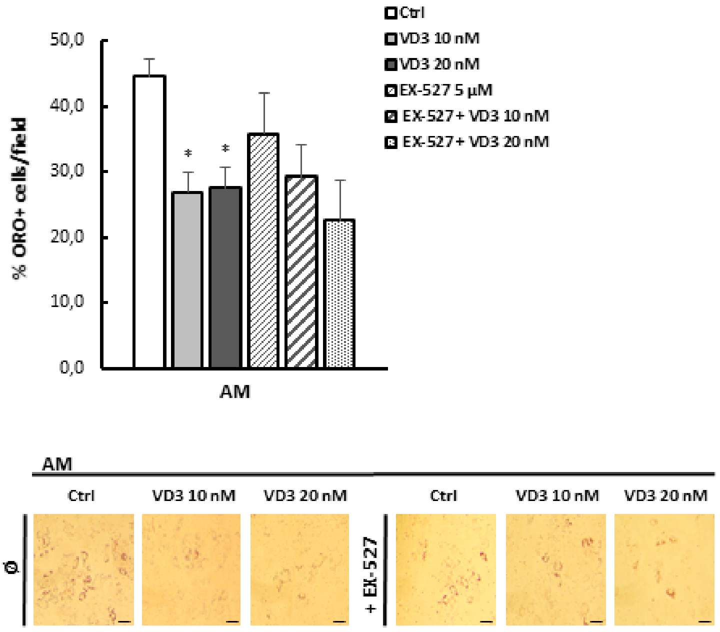

Figure S3. Reduced adipogenic differentiation of VD3-pretreated BM-MSCs: role of SIRT1 signaling. BM-MSCs were pretreated for 5 days with VD3 (10nM or 20nM), EX-527 (5µM) or both in GM. Afterwards, cells were cultured in AM for appropriate period to induce adipogenesis. Intracellular lipid droplets were detected after 28 days cultivation in AM by Oil Red O staining. Representative images are shown (Scale bars: 20µm). Graphs represent quantitative analysis of Oil Red O positive (ORO+) cells percentage enumerated per visual field. Results are presented as means ± SEM for at least three independent experiments. Statistically significant differences by t test: *$p < 0.05$ compared to untreated AM control.
