# Supplementary figures and images for: Vitamin D3 Stimulates Proliferation Capacity, Expression of Pluripotency Markers, and Osteogenesis of Human Bone Marrow Mesenchymal Stromal/Stem Cells, Partly through SIRT1 Signaling
Source: Biomolecules. 2022 Feb 18;12(2):323. doi: 10.3390/biom12020323 (PMC8868595; doi:10.3390/biom12020323)

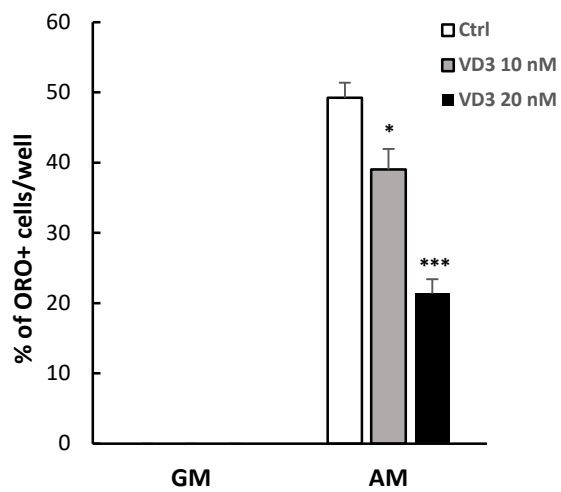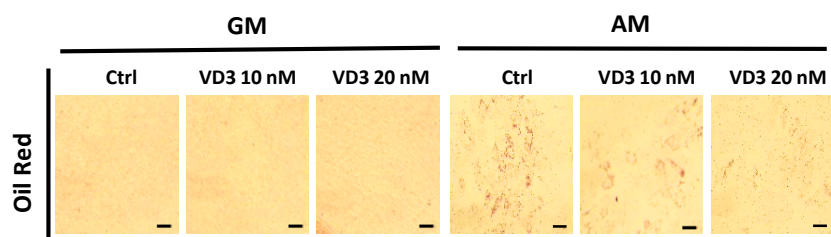

Supplement: Supplementary file 1 [file biomolecules-12-00323-s001.zip › Figure S1.pdf]

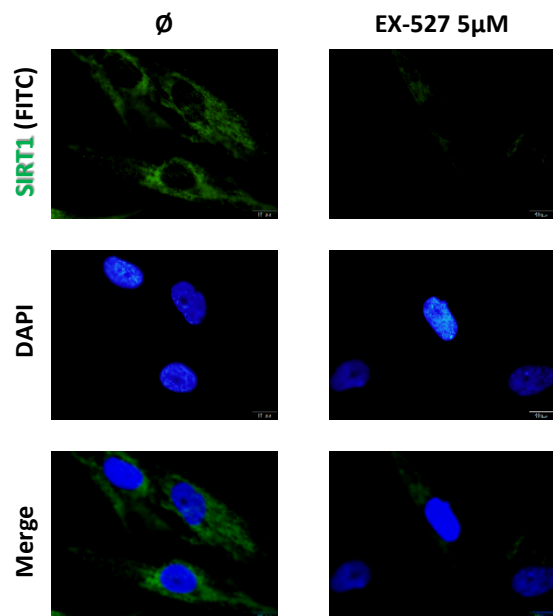

Supplement: Supplementary file 1 [file biomolecules-12-00323-s001.zip › Figure S2.pdf]

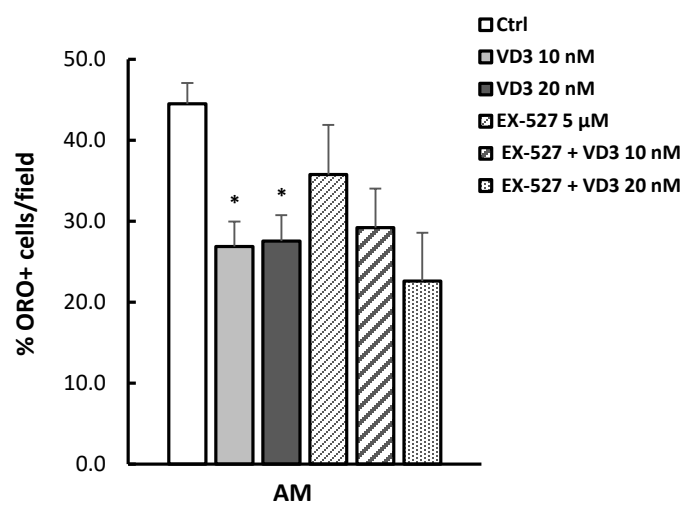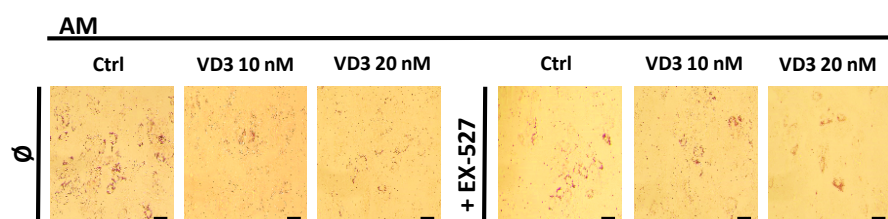

Supplement: Supplementary file 1 [file biomolecules-12-00323-s001.zip › Figure S3.pdf]
